# Application of a small molecule calcium influx inducer as a vaccine adjuvant: enhancing Th2-biased immune responses

**DOI:** 10.3389/fimmu.2026.1704416

**Published:** 2026-02-17

**Authors:** Yumi Yokoyama, Yukiya Sako, Shiyin Yao, Fernando Gil, Renna Cozza, Jasmine Jin, Ian Mclaughlin, Paola Anguiano Quiroz, Tyler Brown, Nikunj M. Shukla, Michael Chan, Maripat Corr, Dennis A. Carson, Tomoko Hayashi

**Affiliations:** Department of Medicine, University of California, San Diego, La Jolla, CA, United States

**Keywords:** adjuvant, Ca2+ influx, dendritic cell, extracellular vesicle, T helper 2, vaccine

## Abstract

**Introduction:**

Vaccines are highly effective in preventing the spread of communicable diseases and are critical to overall public health. As immune stimulants vaccine adjuvants augment the level and longevity of these protective responses. Seeking novel adjuvants using parallel high throughput screens and subsequent systematic structure–activity relationship studies we identified an analogue of a hit compound, **2G272**, that in screening assays retained *in vitro* induction of calcium (Ca^2+^) influx, tetraspanin CD63 EV reporter activity and cell viability. Here, we further our analyses of the biological activity of **2G272** related its potential use as a vaccine adjuvant.

**Methods:**

**2G272** was tested for activation of murine bone marrow-derived dendritic cells (mBMDC) by flow cytometry for Ca^2+^ entry, levels of CD80 and CD86 expression, and *in vitro* stimulation of antigen-specific T cell proliferation. Cytokines and IgG responses from BALB/c mice injected with **2G272** as a single agent or as an adjuvant with ovalbumen were measured by ELISA.

**Results:**

**2G272** triggered store-operated Ca^2+^ entry in mBMDC as well as increases in CD80 and CD86 surface expression. In co-culture experiments, this compound amplified the stimulation of cognate T cell proliferation. Intramuscular injection of **2G272** elicited minimal systemic cytokine and chemokine release. When used as an adjuvant with ovalbumen, **2G272** generated a significant antigen-specific IgG1 response with a higher splenocyte T helper 2 (Th2) cytokine response.

**Discussion:**

**2G272** activated mBMDCs associated with EV release and a store-operated calcium entry response. Enhanced cognate T cell proliferation was mediated either through direct engagement with compound-stimulated mBMDCs or indirectly via immunostimulatory extracellular vesicles released by **2G272**-activated mBMDCs. **2G272** elicited minimal systemic cytokine and chemokine release, demonstrating a promising safety profile. When used as an adjuvant in a murine vaccination model, **2G272** enhanced the IgG1 response with an associated T helper 2 cytokine profile. Hence this compound shows promise as an adjuvant if a Th2 response is beneficial or in combination with other agents to provide a balanced immune response in vaccines.

## 1 Introduction

Vaccines have benefitted both individuals and populations by generating protective immunity to potentially lethal pathogens. Historically aluminum-based adjuvants have been the most widely used and these promote humoral immunity by inducing a Th2 response resulting in IgG1 predominance accompanied by T cell IL-4 and IL-5 production (1–3). Recently, there have been advances in emulsion-based adjuvants (e.g., MF59) (4), nucleic acid adjuvants (e.g., CpG oligonucleotides) and other innate immune stimulants (e.g., monophosphoryl lipid A, MPLA) (5) that have attained FDA approval for human use (6, 7). With the emergence of new pathogenic strains of viruses, the demands for more effective adjuvants that can be used alone or in combination.

To address the need for additional adjuvants that could complement and strengthen the responses to existing adjuvants we undertook three parallel high throughput screening (HTS) campaigns using human reporter cell lines targeting CD63 expression [a marker for extracellular vesicle (EV) release], NF-κB-signaling and interferon-sensitive response element (ISRE) signaling (8). Among 80 hit compounds we identified ethyl 2-(benzo[*c*][1,2,5]thiadiazole-4-sulfonamido)-4,5-dimethylthiophene-3-carboxylate (compound **634**) as a potent inducer of Ca^2+^ influx and EV release (8). Calcium mobilization is a common feature of receptor-mediated cell activation and might be useful as a complementary mechanism to existing adjuvants in promoting effective immune responses (9, 10). Additionally, EVs are a critical component of extracellular communication that could further promote immune activation (11–14) and coupled with Ca^2+^ influx modulation could further augment adjuvant potency.

**Figure 1:**
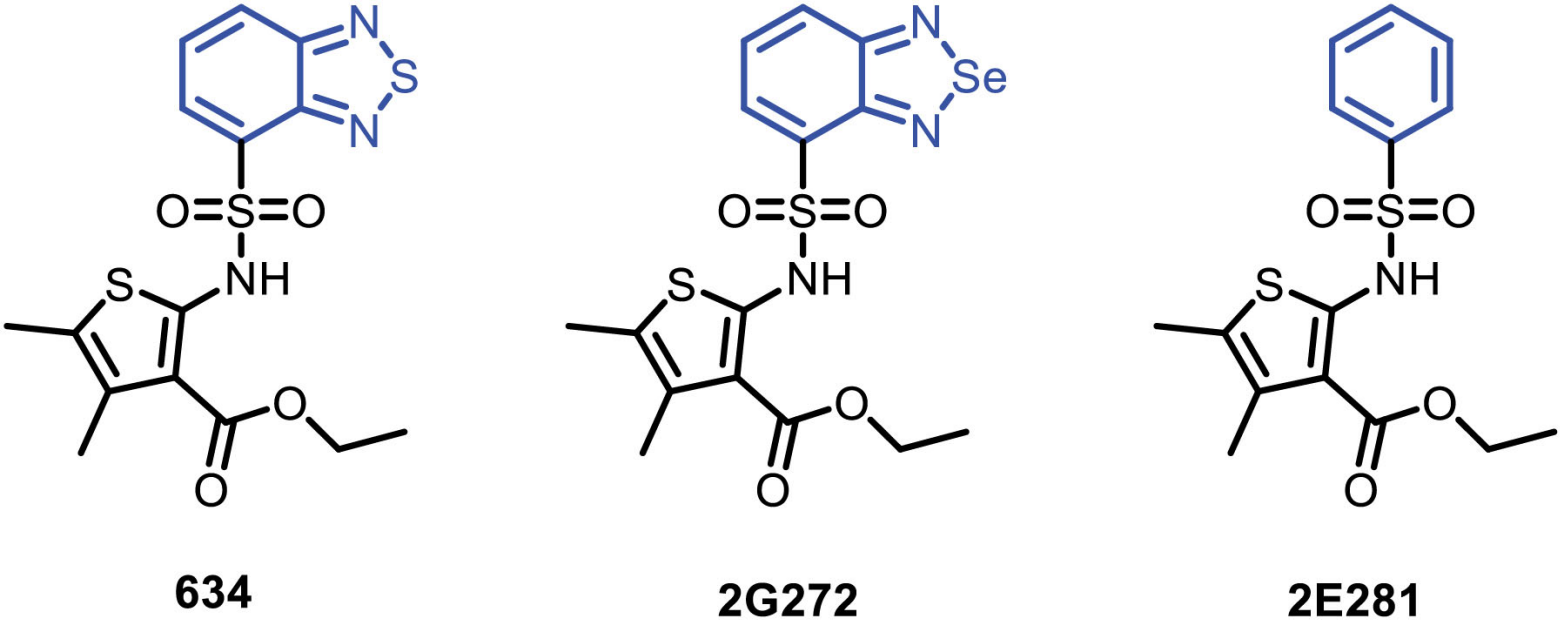
Chemical structures. Shown are the structures for compounds **634**; ethyl 2-(benzo[*c*][1,2,5]thiadiazole-4-sulfonamido)-4,5-dimethylthiophene-3-carboxylate, **2G272**; ethyl 2-(b3enzo[*c*][1,2,5]selenadiazole-4-sulfonamido)-4,5-dimethylthiophene-3-carboxylate, and **2E281**; ethyl 4,5-dimethyl-2-(phenylsulfonamido)thiophene-3-carboxylate (8).

EVs derived from different antigen presenting cells (APCs) including dendritic cells (DCs) and B lymphocytes have been used to induce antigen-specific MHC class II-restricted T cell responses in human and murine systems (11, 15). The EVs released from murine bone marrow derived dendritic cells (mBMDC) treated with compound **634** expressed increased co-stimulatory molecules, CD80 and CD86, and promoted T cell activation, proliferation, and cytokine release demonstrating immunopotency (8, 16). Subsequently, we conducted structure-activity relationship studies (SAR) with 59 analogs using *in vitro* screens for Ca^2+^ influx, tetraspanin CD63-reporter gene expression and cell viability in mBMDC and preliminary *in vivo* adjuvanticity screening (16). The SAR studies indicated that analogs of compound **634** that retained the ester functional group and had selected bicyclic ring connected to sulfonyl moiety retained activity. Of all the active analogs, we identified that the replacement of sulfur atom in the benzothiadizole ring with selenium provided a potent selenediazole compound **2G272**, while removing the thiadiazole functional group to obtain monocyclic ring analog **2E281** led to loss of activity (**Figure 1**) (16). Here we further characterize **2G272** as a lead compound, evaluating its ability to induce Ca^2+^ influx and its immunostimulatory efficacy *in vitro* and *in vivo*.

## 2 Materials and methods

### 2.1 Chemical synthesis

Compounds **2G272** and **2E281** were synthesized in our laboratory (8, 16). The reagents were purchased as reagent grade from commercial vendors unless otherwise specified and used without further purification. Solvents were purchased from Fisher Scientific (Waltham, MA) and were either used as purchased or redistilled with an appropriate drying agent. Instrumentation, synthesis, and NMR analysis of **2G272** and **2E281** were previously published and correspond to compound IDs 9f and 9j, respectively (16). The purity of compounds **2G272** and **2E281** was consistently >95%.

### 2.2 Animals

Six to eight-week-old female BALB/c mice (RRID: IMSR_JAX:000651) and major histocompatibility (MHC) class II restricted ovalbumin T cell receptor transgenic mice DO11.10 strain (RRID: IMSR_JAX:003303) (17) were purchased from the Jackson Laboratory. The animal husbandry was performed by UC San Diego Animal Care Program, including 24 hour veterinary care support. All animal experiment protocols in this study received prior approval by the Institutional Animal Care and Use Committee (IACUC) for UC San Diego (approved protocol number S00028).

### 2.3 Cell culture

Murine bone marrow-derived dendritic cells (mBMDCs) were prepared from bone marrow harvested from femurs and tibia of BALB/c mice as previously described (18, 19). Studies were performed in RPMI-1640 supplemented with 10% heat-inactivated Fetal bovine serum (FBS) and 100 U/mL Penicillin/Streptomycin (RP10) in humidified conditions with 5% CO_2_ at 37˚C. Detailed information on tissue culture reagents is provided in **Supplementary Table S2**.

### 2.4 Ca^2+^ influx assay

Ca^2+^ influx was measured using Fura-8-AM reagents. mBMDCs were loaded with ratiometric Ca^2+^ indicator Fura-8-AM (4 µM) in Hank’s Balanced Salt Solution (HBSS) assay buffer [1 × HBSS, 10 mM HEPES (pH 7.4), 1.8 mM CaCl_2_, 0.8 mM MgCl_2_, and 0.1% BSA] containing 0.04% Pluronic F127 at 37 ˚C for 40 min and at RT for additional 20 min. OD_355nm_ and OD_415nm_ (excitations) and OD_540nm_ (emission) were read and test compounds were added at the indicated time. OD ratios for 355 nm/415 nm were used to assess changes in intracellular Ca^2+^ by a fluorescence plate reader (Tecan Spark, TECAN, San Jose, CA).

### 2.5 Ca^2+^ add-back assay

mBMDCs were incubated with Fura-8-AM in Ca^2+^ depleted HBSS assay buffer [1 × HBSS, 10 mM HEPES (pH 7.4), 0.8 mM MgCl_2_, and 0.1% BSA] containing 0.04% Pluronic F127 at 37 °C for 40 min and the compound was added at the indicated time point. OD_355/415_ (excitations) and OD_540_ (emission) were read for 10 min. Ca^2+^ was added to a final concentration of 1.8 mM, and OD_355/415_ and OD_540_ were monitored for an additional 20 min. Data are presented as OD ratios for 355nm/415nm.

#### 2.5.1 Costimulatory molecule expression analysis

Costimulatory molecule expression on mBMDC was measured by the flow cytometry assay as described previously (28). mBMDCs (10^6^ cells/mL) were incubated with 10 μM compound, 1 μM Ionomycin (ION) or 1 μg/mL Monophosphoryl lipid A (MPLA) overnight. Dimethyl sulfoxide (DMSO, 0.5%) was used as the vehicle. Cells were incubated with anti-mouse CD16/32 antibodies for blocking FcR and stained with anti-CD11c, anti-CD80 or anti-CD86 antibodies (listed in **Supplementary Table S1**) for 30 min at 4 °C. Cells were stained with 4′,6-diamino-2-phenylindole (DAPI) for 10 min at RT to exclude dead cells. Data were acquired using MACSQuant flowcytometer and analyzed with FlowJo (version 10.10.0, Becton Dickinson, Ashland, OR). The gating strategy is shown in **Supplementary Figure S1**.

### 2.6 EV Isolation by differential ultracentrifugation

EVs were isolated from the culture media of the test compound-stimulated mBMDC as described (20). mBMDCs were cultured with 10 µM **2G272**, **2E281**, or 1 µM ION or DMSO (0.25 %) for 48 hours in RP10 media. Conditioned culture medium (40 mL) was spun at 300 x *g* for 10 min, at 2000 x *g* for another 10 min, and at 10,000 x *g* for 30 min. Next, 30 mL of supernatant was transferred to 31.5 polypropylene ultracentrifugation tubes and spun at 100,000 x *g* (average) for 3 hours in an SW28 rotor (K-factor: 2554) by a Beckman Optima XL-90 ultracentrifuge (Beckman Coulter Life Sciences, Brea, CA). The supernatant was aspirated (leaving ∼50 μL), and the pellet was resuspended in 30 mL PBS. The resuspended pellet was spun under the same conditions as the previous spin, followed by another round of gentle aspiration and resuspension to a final volume of 50 μL in cold 0.02 μm filtered PBS. All centrifugation steps were performed at 4 °C, and the resulting samples were stored at −80 °C until use. All relevant data was submitted to the EV-TRACK knowledge base (EV-TRACK ID: EV250096).

### 2.7 Transmission electron microscopy

The morphologies of the harvested EVs were imaged by transmission electron microscopy by the Nanoparticle Characterization Laboratory (Frederick National Laboratory for Cancer Research, Frederick, MD). The sample (3 μL) was applied to a glow discharged lacey carbon film grid (Electron Microscopy Sciences, Reutlingen, Germany) and frozen using an FEI Vitrobot at 95% humidity, with a blot time of 5 seconds and a blot force of 0. Images were taken using a T20 transmission electron microscope (Thermo Fisher Scientific) equipped with Gatan Rio camera (Pleasanton, CA).

### 2.8 Immunoblotting

Immunoblotting was performed using anti-CD81, anti-Alix, anti-CD80, and anti-CD86 antibodies as primary antibodies. The total protein in the EV samples was quantitated by the Micro BCA Assay kit. Two (CD80 and CD86) or four (CD81 and Alix) micrograms of protein of EV lysates were mixed with 4× NuPAGE sample buffer under reducing conditions with dithiothreitol (DTT). Two µg/well was loaded for CD80 and CD86, and 4 µg/well was loaded for CD81 and Alix blots. Samples were denatured at 95 °C for 5 min prior to loading. After fractionation on NuPAGE 4−12% Bis-Tris gels, proteins were blotted onto Immobilon-P PVDF membranes and blocked for 1 hour in 5% BSA TBS with 0.1% Tween 20 at room temperature (RT). The blots were then incubated with primary antibodies (Abs) (1:1000 dilution), overnight at 4 °C with gentle agitation. After washing, the membranes were incubated with the corresponding secondary antibody for 30 min at RT with gentle agitation. Blots were developed with SuperSignal™ West Dura Extended Duration Substrate and visualized using a ChemiDoc Imaging System. AccuRuler Prestained Protein Ladder was used for the molecular weight markers. Details for antibodies and reagents are listed in **Supplementary Table S1**.

### 2.9 Antigen-specific T cell proliferation assay

mBMDCs from BALB/c mice were treated with vehicle (0.5% DMSO), 10 µM **2G272**, 10 µM **2E281**, or 1 µM MPLA overnight and then the BMDCs were pulsed with OVA protein for 4 hours. After washing with media twice, the OVA-pulsed BMDCs or unpulsed BMDCs were cocultured with carboxyfluorescein succinimidyl ester (CFSE)-labeled splenic CD4^+^ T cells isolated from DO11.10 mice, which are transgenic for T cell receptor (TCR) specific for I-A^d^ with OVA_323–339_ peptide. Splenic CD4^+^ T cells were isolated using EasySep Mouse CD4^+^ T cell isolation kit (negative selection). For the EV induced antigen-specific T cell proliferation assay, CFSE (2 μM)-labeled DO11.10 CD4^+^ T cells were cocultured with an equal volume of EVs (20 µg) in the presence of OVA_323−339_ peptide. After a 5-day incubation, cells were stained with anti-mouse CD4 or anti-mouse DO11.10 clonotypic TCR antibody and antigen-specific CD4^+^ T cell proliferation was evaluated by CFSE fluorescence using a MACSQuant flow cytometer. Data were analyzed using FlowJo software (FlowJo; version 10.8.1, FlowJo, Ashland, OR). IL-2 in the supernatant was measured using a Mouse IL-2 DuoSet ELISA kit. Details regarding antibodies are listed in **Supplementary Table S2**. The gating strategy is shown in **Supplementary Figure S1**.

### 2.10 *In vitro* cytokine measurement

Cytokine levels in culture supernatants were harvested and tested for cytokines by ELISA. Antibodies and kits are listed in **Supplementary Table S2**.

### 2.11 *In vivo* systemic cytokine release study

BALB/c mice were *i.m.* injected with **2G272** (200 nmol/animal) or vehicle (0.5% DMSO) in two limbs. MF59, ION and MPLA were used as controls. Sera samples were collected at 8, and 48 hours after injection. IL-6, IL-12, CXCL10, CXCL1, CCL2, CCL5, TNF and alpha-1 acid glycoprotein were measured by ELISA and Luminex bead assays. Detection limits of these assays are described in **Supplementary Table S3**.

### 2.12 *In vivo* immunization protocol

OVA (5 µg/animal) was mixed with a test compound (200 nmol/animal), MPLA (1 µg/animal) or MF59 (25% solution). The mice were immunized on day 0 and 21 and sera were collected on day 28 and 42. Vehicle (0.5% DMSO) served as a negative control. 50 μL of each agent was injected into the gastrocnemius muscle using a 29G insulin syringe. Immunization schedules and sample size are indicated in each figure legend. OVA-specific IgG2a and IgG1 titers were measured by ELISA as previously described (21). For antigen-specific splenocyte recall responses, spleens were harvested on day 42, and splenocytes (1×10^6^ cells/well/0.2mL) were re-stimulated with 100 μg/mL OVA for 5 days. IL-5 and IFNγ were selected as robust and reproducible assays on previously frozen supernatants and were measured by ELISA. Anti-OVA IgG1 and IgG2a were measured by ELISA. Assays were performed using half area 96 well plates as previously described (22). In brief, plates were coated with antigens, blocked, then incubated with serially diluted sera. After incubation at 4 °C or RT overnight, plates were washed, and the detection antibodies were added. After washing and incubation with p-nitrophenyl phosphate substrate (p-NPP), plates were read at 405 nm and 570 nm as a reference on TECAN plate reader. We prepared standard sera with known endpoint titers as previously reported (22). The titer of the test sample was interpolated from the standard serum titration from the same plate. The results are expressed in units per milliliter (arbitrary units). Detailed information on reagents and antibodies is provided in **Supplementary Table S2**.

### 2.13 Statistical analyses

To compare multiple groups, one-way ANOVA with Dunnett’s *post hoc* test or two-way ANOVA with Tukey’s *post-hoc* test were used for *in vitro* assays. For *in vivo* nonparametric data the Kruskal-Wallis test with Dunn’s *post-hoc* test was used. Prism 10 software (GraphPad Software, San Diego, CA) was used. P values < 0.05 were considered statistically significant.

## 3 Results

### 3.1 Activation of murine dendritic cells by **2G272** is dependent on Ca^2+^ influx

**Figure 2:**
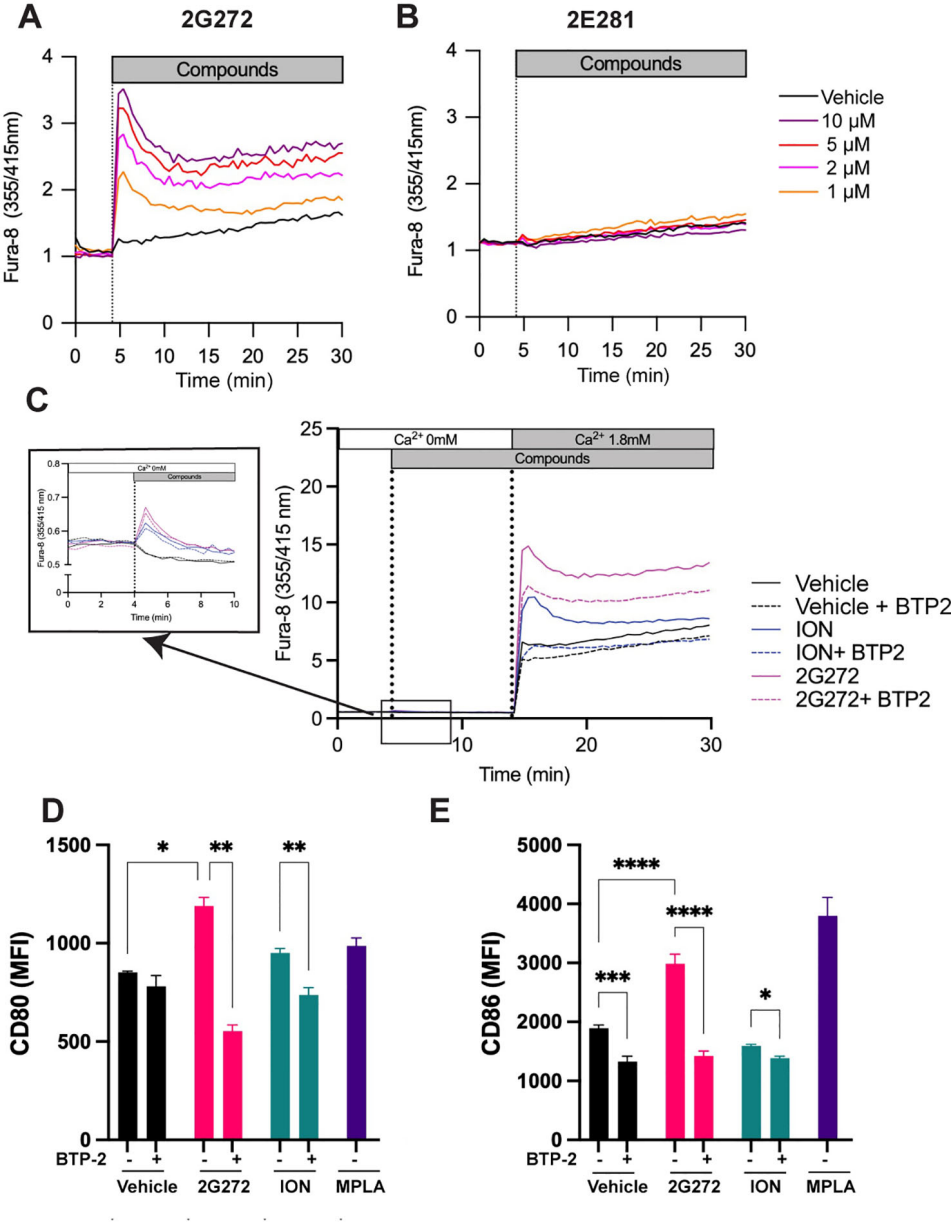
**2G272** increases intracellular Ca^2+^ levels associated with APC functions. **(A, B)** Calcium mobilization in mBMDCs treated with **2G272** (A), or **2E281 (B)**. mBMDCs were loaded with Fura-8 and stimulated with 1, 2, 5 and 10 µM of the indicated compound. The time-course of intracellular Ca^2+^ levels was recorded for 25 minutes after stimulation. The dashed line indicates the addition of compound. Representative data from two independent experiments with similar results are shown. **(C)** Ca^2+^ restoration assay to assess SOCE in mBMDCs. Fura-8-loaded mBMDCs were treated with ION (1 µM), **2G272** (10 µM) alone or in combination with 10 µM BTP2 (SOCE inhibitor) for 10 minutes in Ca^2+^ free medium. Subsequently, Ca^2+^ was added (final concentration: 1.8 mM), and the resulting Ca^2+^ influx was measured. Representative data from two independent experiments with similar results are shown. **(D, E)** Expression of costimulatory molecules, CD80 **(D)** and CD86 **(E)** were measured by flow cytometry 24 hours after treatment with **2G272** (10 µM), **2E281** (10 µM), or MPLA (1 µg/ml) in the presence or absence of BTP2 (10 µM). *p < 0.05, **p < 0.01, ***p < 0.001, ****p < 0.0001, by two-way ANOVA with Tukey’s *post-hoc* test. Representative data from two independent experiments with similar results are shown.

We previously demonstrated that a hit compound from the HTS, **634**, activates SOCE dependent Ca^2+^ influx and can activate murine bone marrow derived dendritic cells (mBMDCs) (8). To determine whether the derivative **2G272** shares similar functions, we evaluated its effect on intracellular Ca^2+^ levels in mBMDCs using Fura-8 ratiometric Ca^2+^ indicator and measuring the OD_355/415_ ratios. mBMDCs were stimulated with potent compound **2G272** or a low activity analog of the same chemotype, **2E281** (**Figure 1**). **2G272** increased intracellular Ca^2+^ levels in a dose-dependent manner (**Figure 2A**), while **2E281** had no effect on the OD_355/415_ ratios (**Figure 2B**). Notably, in the absence of extracellular Ca^2+^, **2G272** induced a brief and small increase in intracellular Ca^2+^ levels (**Figure 2C** inset), followed by a much larger Ca^2+^ increase upon replenishment of extracellular Ca^2+^ (**Figure 2C**). The intracellular Ca^2+^ kinetics of **2G272** were similar to the kinetics of a control Ca^2+^ ionophore, ION. The SOCE inhibitor, BTP2, depressed the Ca^2+^ influx induced by 1 µM ION to baseline.

As the efficacy of APCs to prime naïve T cells is dependent on the upregulation of costimulatory molecules, we examined CD80 and CD86 expression following treatment of mBMDC with **2G272**. **2G272** increased surface CD80 and CD86 expression by mBMDCs (**Figures 2D, E**; the gating strategy shown in **Supplementary Figure S1A**) compared to vehicle treated cells (p < 0.0001). Notably these increases returned to baseline levels by co-incubation with BTP2, an inhibitor of SOCE-mediated Ca^2+^ influx (p < 0.0001; **Figures 2D, E**). MPLA, an FDA-approved vaccine adjuvant (5), served as a comparative stimulator for mBMDCs. These results highlight that **2G272** activation of certain antigen-presenting functions are largely dependent on Ca^2+^ influx.

### 3.2 **2G272** enhances antigen presenting cell stimulated T cell proliferation

**Figure 3:**
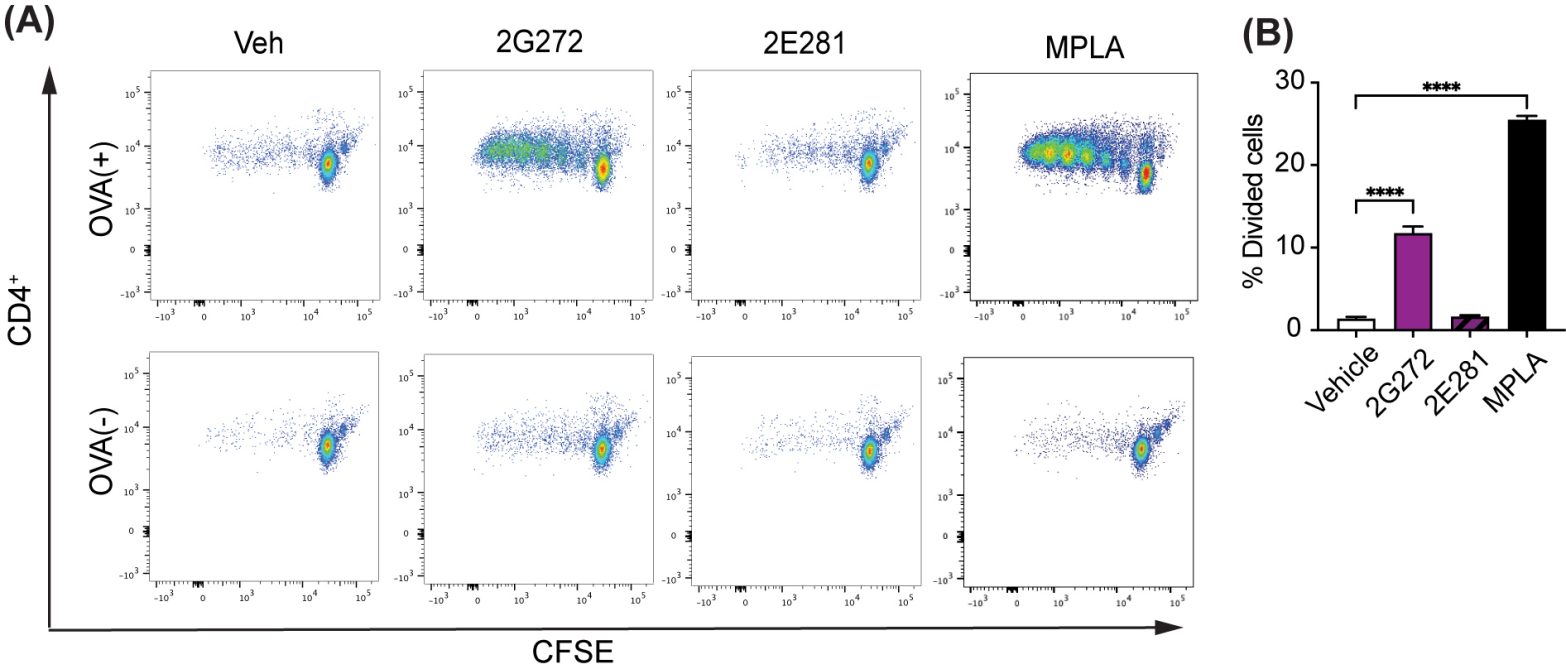
Immunomodulatory effects of **2G272** on dendritic cells and T cell proliferation. **(A, B)** BMDCs from BALB/c mice were treated with vehicle (0.5% DMSO), 10 µM **2G272**, 10 µM **2E281**, or 1 µM MPLA overnight. Following a 4-hour incubation with OVA protein, CFSE-labeled CD4^+^ T cells from DO11.10 mice were added to mBMDCs and incubated for 5 days. **(A)** Dot plots of CD4^+^ T cell division by CSFE generational dilution. **(B)** Percentage of divided CD4^+^ T cells. ****p < 0.0001, by ordinary one-way ANOVA with Dunnett’s *post-hoc* test. Representative data from two independent experiments with similar results are shown.

To investigate whether **2G272** potentiates APC T cell priming and stimulation, we performed a differentiated dendritic cell (DC) and T cell co-culture study (18). Briefly, mBMDCs were incubated overnight with **2G272**, **2E281**, or MPLA, and OVA protein was then added for the last 4 hours. The cells were washed and co-cultured with carboxyfluorescein succinimidyl ester (CFSE)-labeled OVA specific transgenic DO11.10 CD4^+^ T cells. T cell receptor (TCR)-dependent T cell proliferation was monitored by CFSE dilution after five days using flow cytometry (**Figures 3A, B**; gating strategy shown in **Supplementary Figure S1B**). Notably, mBMDCs treated with **2G272** significantly enhanced CD4^+^ T cell proliferation, while the low activity analog **2E281** had minimal effects on T cell proliferation. These findings demonstrate that **2G272** activates APCs thereby promoting TCR dependent T cell priming and proliferation.

### 3.3 **2G272** promotes the release of immunostimulatory EVs

**Figure 4:**
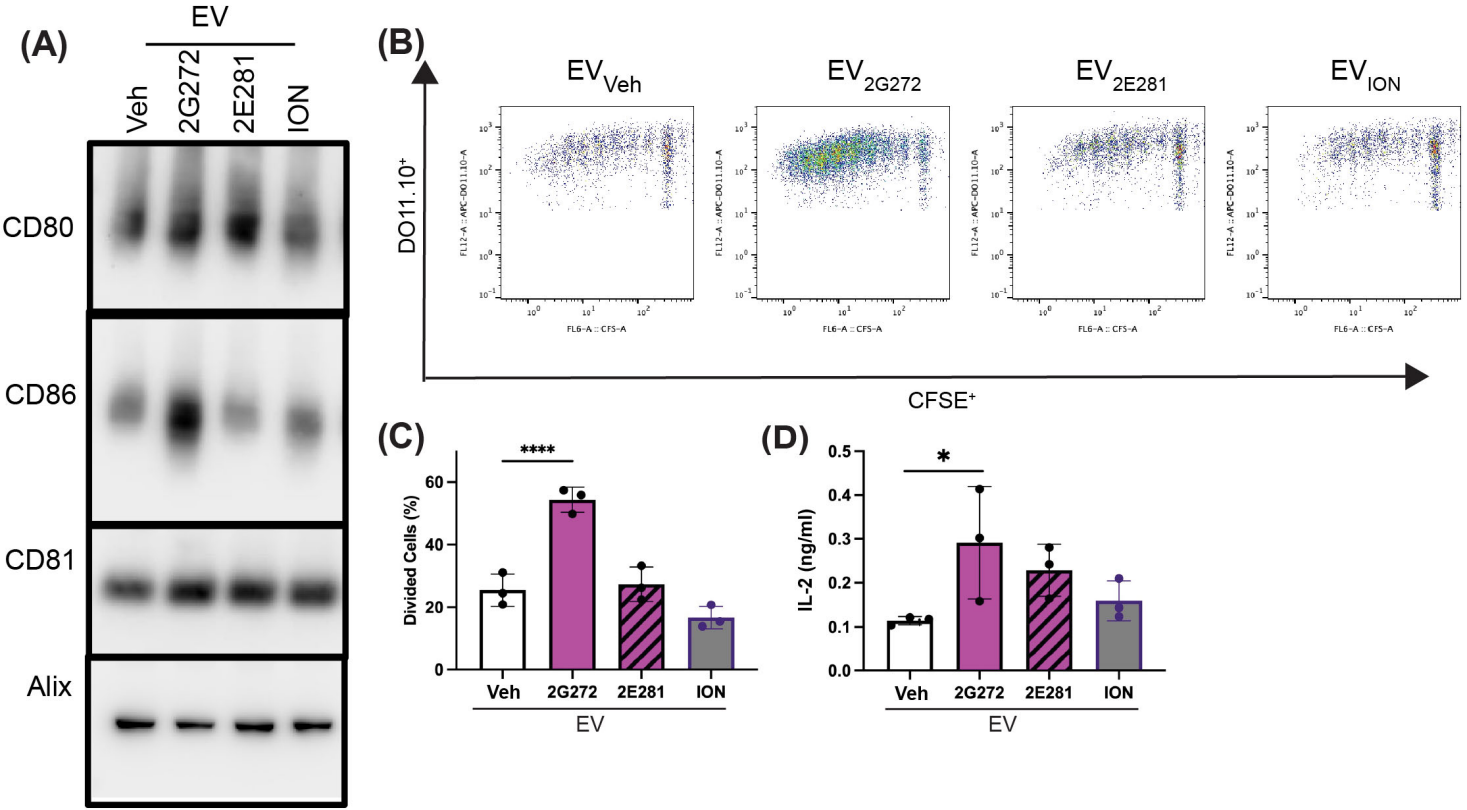
Compound **2G272** promotes the release of immune-stimulatory EVs from mBMDCs. **(A)** Characterization of EVs by immunoblotting. mBMDCs were treated with vehicle (Veh, 0.25% DMSO), **2G272** (10 μM), **2E281** (10 μM), or ION (1 μM) overnight. EVs from the supernatant were harvested, lysed and proteins (2 µg/well for CD80 and CD86, 4 μg/well for CD81 and Alix) were separated by electrophoresis and immunoblotted as shown. Full blots are shown in **Supplementary Figure S2**. **(B)** EV_**2G272**_ enhances T cell proliferation in the presence of antigenic peptides. CFSE-labeled CD4^+^ T cells from transgenic DO11.10 mice were treated with 20µg in equal volumes of EV_**2G272**_, EV_**2E281**_, EV_ION_, or EV_Veh_ in the presence or absence of OVA_323–339_ peptide for 5 days. T cell proliferation was determined by CFSE dilution using flow cytometry, shown as dot plots and **(C)** percentages of divided T cells. **(D)** IL-2 concentrations in the culture supernatants at day 5 were measured by ELISA. Data are means ± SDs of triplicates, representative of two independent experiments. *p < 0.05, ****p < 0.0001 by ordinary one-way ANOVA and Dunnett’s *post-hoc* test compared to EV_Veh_.

Calcium signaling regulates APC function and their EV release (15, 23). We previously reported that the parent hit compound **634** induced the production of immunostimulatory EVs and its SAR analog **2G272** stimulated CD63 reporter activity as a surrogate marker of EV biogenesis. To expand on the CD63 reporter assay, we examined if **2G272** could stimulate the production of functionally active EVs. The EVs released from **2G272** (EV_**2G272**_), **2E281** (EV_**2E281**_), ION (EV_ION_) or vehicle (0.25% DMSO, EV_Veh_) treated mBMDCs were harvested by differential ultracentrifugation. The tetraspanin CD81, and Alix were detected in all isolated EVs (**Figure 4A** and **Supplementary Figure S2**) and the morphologies of EV_**2G272**_ and EV_Veh_ were validated by CryoTEM (Nanoparticle Characterization Laboratory; **Supplementary Figure S3**). Only EV_**2G272**_ demonstrated an increase in both CD80 and CD86 expression, while EV_**2E281**_ showed a minor increase in CD80 expression by immunoblotting (**Figure 4A**).

We then examined the antigen-presenting function of EVs released from mBMDCs stimulated with **2G272** or **2E281** (**Figures 4B-D**) using DC-T cell-co-culture experiment. CFSE-labeled OVA specific DO11.10 CD4^+^ T cells were cocultured with equal numbers of EVs in the presence of OVA_323−329_ peptide for five days. In this assay the MHC II on the surface of EVs were loaded with extracellular OVA_323−329_ peptide as they cannot process whole protein into specific peptides. EV_**2G272**_ enhanced peptide specific T cell proliferation, as demonstrated by the increase in the diluted CSFE populations (**Figure 4B**; gating strategy shown in **Supplementary Figure S1C**) and the percentage of divided cells in the presence of EV_**2G272**_ (**Figure 4C**), but not EV_**2E281**_. Furthermore, the supernatant from the EV_**2G272**_ co-culture showed elevated levels of IL-2, a cytokine essential for T cell proliferation (**Figure 4D**). These data collectively demonstrate that EVs released by **2G272**-treated-mBMDC are sufficient to stimulate proliferation of TCR-dependent antigen-specific T cells.

### 3.4 *In vivo* administration of **2G272** does not stimulate systemic cytokine release

**Figure 5:**
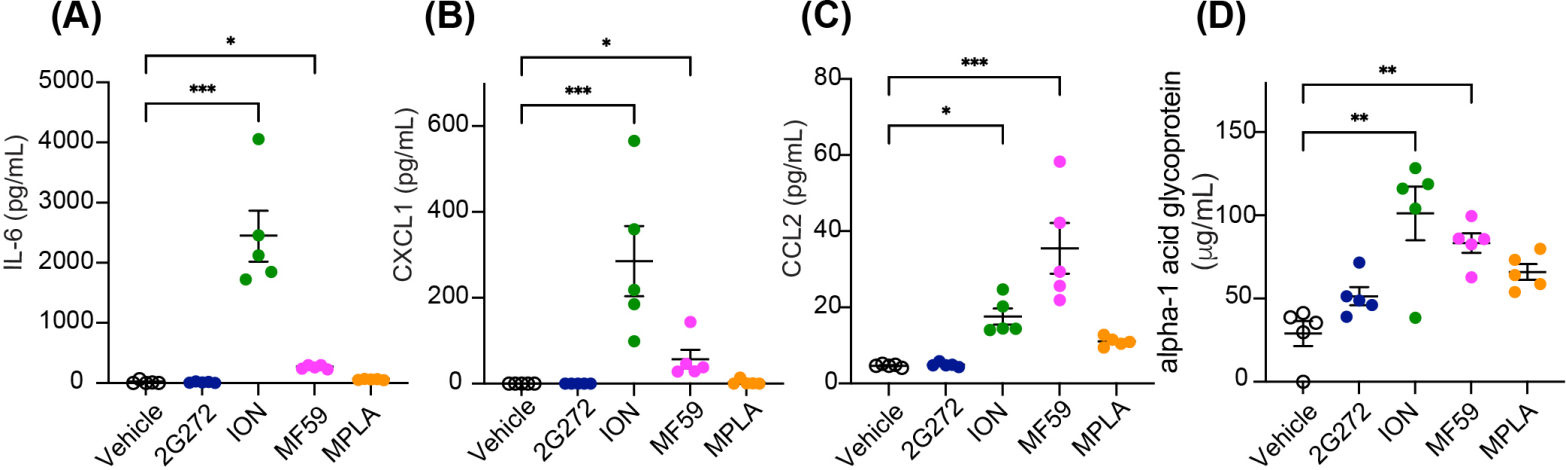
Cytokine, chemokine and inflammatory responses in mice administered with **2G272**. BALB/c mice (n=5/group) were *i.m.* injected with **2G272** (200 nmol/animal) or vehicle (0.5% DMSO). MF59 (25 µL/animal) was used as a positive control. Sera samples were collected 8, or 48, hours after injection. The levels of **(A)** IL-6 (8 hours), **(B)** CXCL1 (8 hours), **(C)** CCL2 (8 hours), and **(D)** alpha 1 acid glycoprotein (48 hours) were measured in serum samples using ELISA and Luminex bead assays. Serum concentrations of IL-12, TNF, CCL5 and CXCL10 were below detectable levels. Statistical significance was determined by Kruskal-Wallis test followed by Dunn’s *post-hoc* test compared to the MF59 control: ***p < 0.001, **p < 0.01, and *p < 0.05.

We conducted a comprehensive analysis of serum cytokines and chemokines in BALB/c mice after intramuscular (*i.m*.) injection of **2G272**. **2G272** (200 nmol/animal) was administered to mice and sera were collected at 8 or 48 hours post-administration. MF59, the adjuvant for the FDA-approved vaccine used in FLUAD^®^ (24), ION, and MPLA were used as comparators. Subsequently levels of IL-6, CXCL1 and CCL2 and alpha-1 acid glycoprotein were compared to those by ION, MF59, and MPLA in sera. **2G272** did not induce cytokines, chemokines, and inflammatory proteins, while ION and MF59 produced significantly higher IL-6, CXCL1, CCL2, and alpha-1 acid glycoprotein (**Figure 5**). These findings suggest that **2G272** presents minimal systemic reactogenicity compared to ION and MF59.

### 3.5 **2G272** as adjuvant induces Th2-biased immune responses *in vivo*

**Figure 6:**
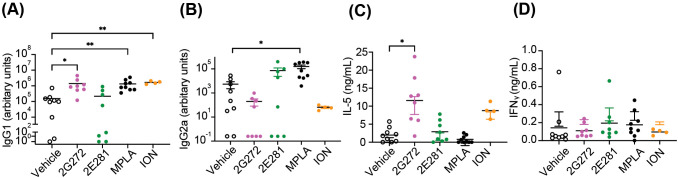
**2G272** elicits Th2 biased immune responses in a murine model. **(A, B)** OVA-specific IgG1 and IgG2a antibody responses. Female BALB/c mice (n=4-9) were immunized with OVA (5 µg/animal) adjuvanted with **2G272** (200 nmol/animal), **2E281** (200 nmol/animal), MPLA (1 µg/animal), or ION (200 nmol/animal) on days 0 and 21. Serum samples were collected on day 28 and OVA-specific IgG1 and IgG2a levels were determined by ELISA. **(C, D)** On day 42, splenocytes were cultured with OVA for 5 days, and IL-5 **(C)** and IFN-γ **(D)** in the supernatant were measured by ELISA. Data are presented as individual values and as means ± SEM. Data shown are pooled from two experiments. Statistical significance was determined by Kruskal-Wallis test followed by Dunn’s *post-hoc* test compared to the vehicle control: **p < 0.01, *p < 0.05.

To evaluate the *in vivo* adjuvant activity of **2G272**, we immunized BALB/c mice with OVA protein in the presence and absence of **2G272** and compared the results to those obtained with the SOCE-dependent Ca^2+^ inducer ION, and to MPLA, which is known for promoting both Th1/Th2 responses (**Figure 6**). The lower activity analog **2E281** and the vehicle served as negative controls. Sera were collected on day 28, and OVA-specific immunoglobulins (IgG1 and IgG2a) were measured by ELISA (**Figures 6A, B**). Splenocytes were cultured with OVA to assess antigen-specific T cell responses. **2G272**-adjuvanted vaccine induced significantly higher levels of antigen-specific IgG1 compared to the vehicle-only adjuvanted vaccine (p < 0.02), both showing minimal levels of IgG2a. ION-adjuvanted vaccine induced high levels of IgG1 and minimal levels of IgG2a, like that of **2G272**. As expected, the MPLA-adjuvanted vaccine induced high levels of both IgG1 and IgG2a (p = 0.008 and 0.02, respectively) (**Figures 6A, B**). Furthermore, splenocytes from mice immunized with **2G272**-adjuvanted OVA released significantly higher levels of IL-5 (p < 0.05), a robust signature cytokine of Th2-biased immune responses (25, 26), but not IFNγ, which mirrored the isotypes of the antibody data (**Figures 6C, D**).

## 4 Discussion

Vaccines are a key component of public health and preventing the spread of deleterious viruses. Many effective vaccines contain at least one adjuvant which serves to increase neutralizing antibody titers and enhance cellular immune responses. Adjuvants can effectively steer and enhance specific adaptive immune responses by targeting signaling pathways in innate immune cells, specifically antigen presenting cells. Currently licensed adjuvants for human use include aluminum based adjuvants, MF59 (oil-in-water emulsion), AS01 (liposome with MPLA + QS-21), AS04 (3-deacyl-MPLA), AS03 (vitamin E/Surfactant polysorbate 80/Squalene), and CpG ODN 1018 (an oligonucleotide) (27). These adjuvants are used in a variety of vaccines and work through mechanisms that increase inflammasome activation, or pattern recognition receptors (PRRs) associated signaling (28, 29). Co-adjuvants which trigger complementary signaling pathways may act as amplifiers to the pattern of immunity elicited by the existing adjuvants or could broaden the types of immune responses contributing to improved protective responses (7).

### 4.1 Discovery and characterization of small molecule adjuvants

To identify novel immunomodulatory small molecules that could serve as single or co-adjuvants, we conducted parallel HTS campaigns using overlapping chemical libraries. These human cell-based strategies employed reporter constructs to detect compounds acting through previously unrecognized pathways that converged on immunologically relevant signaling targets (30). Multiple hit compounds were identified that increased secretion of a CD63 fusion reporter product (as a surrogate for EV release), and NF-κB and ISRE signaling that did not compromise *in vitro* cell viability (30). A highly active hit compound, **634**, was selected (8) and SAR studies demonstrated that the relevant activities were associated with preserving the ester group. An analog **2G272** had significant activity in mobilizing Ca^2+^ in primary APCs and increased CD63 reporter activity and was advanced for further study (16). We have not definitively identified the target(s) of compound **2G272**. To assess potential binding targets, we screened **2G272** using an InVEST^®^ panel (Reaction Biology Corp) (31). This panel screens for binding to 53 clinically relevant targets that include select GPCRs, ion channels, biogenic amin transporters, nuclear receptors, phosphodiesterases, cytochrome P450 isozymes (Cyp) and proteases. Binding was detected to cytochrome P isozymes (Cyp3A4, Cyp3A5, and Cyp2C9, Cyp1A2) and D1 dopamine receptor. These findings provide initial insights into the potential safety of **2G272**, but the strength of the interactions indicates that these are not likely to be the primary functional target (31).

### 4.2 **2G272** enhances antigen presentation via dendritic cells and EVs

Using ovalbumin as a test antigen, **2G272** and the calcium ionophore, ION, showed efficacy as single adjuvants (**Figure 6**). Resting immune cells maintain low intracellular Ca^2+^ levels, but upon activation, Ca^2+^ influx from the extracellular space triggers a cascade of intracellular signaling events (32–36). Additionally, extracellular Ca^2+^ has been reported to act as a danger signal activating the NLRP3 inflammasome through G protein-coupled calcium-sensing receptors (9), which stimulates APCs. The increases in CD80 and CD86 on mBMDCs induced by **2G272** were associated with Ca^2+^ modulation as the SOCE inhibitor, BTP2, significantly attenuated the increase in expression. As Ca^2+^ mobilization is a common accompaniment of multiple receptors that activate cells, **2G272** might also be useful as a complementary mechanism in combination adjuvants which warrants additional studies.

Dendritic cells treated with **2G272** increased TCR-dependent antigen-specific T cell stimulation through both direct DC contact and EV mediated mechanisms. EVs facilitate intercellular communication and immune response modulation (8), and are released by a Ca^2+^ dependent mechanism in a stress response (35). The increased surface expression of CD80 and CD86 of **2G272** activated mBMDC was also seen on the EVs shed into the media of treated cells. In addition to costimulatory molecules EVs can carry functional peptide–MHC (pMHC) complexes and directly present antigenic peptides to T cells or can induce phenotypic and functional maturation of dendritic cells (11, 37). One limitation of our study was that the number of EVs were not quantitated for each assay, rather protein equivalents were used. Also, in our assay system the antigen or peptide was provided in the medium; however, plasmacytoid DC-derived EVs can transfer antigen to DCs to enable cross-presentation to naive CD8^+^ T cells (13). While SOCE is required for antigen cross-presentation by DCs (38), we did not formally test cross presentation in either of our co-culture antigen presentation systems. Hence **2G272** can enhance antigen presentation and priming of naïve T cells by APCs either through direct cell-to-cell contact or via EVs released by **2G272** treated APCs.

### 4.3 *In vivo* immune response and safety profile

Using **2G272** as a single adjuvant *in vivo* resulted in T cell production of IL-5 and an IgG1 predominant antibody response consistent with an Th2 profile in BALB/c mice. This pattern aligns with our initial screening strategy as NF-κB is required for DCs to initiate Th2 immune responses (39, 40). In our assays we focused on IL-5 and IFNγ as robust cytokines to assay; however future studies should include IL-4 and IL-13 to further strengthen the phenotype (25, 26). The calcium ionophore ION also stimulated a Th2 related anti-OVA immune response indicating that Ca^2+^ influx may be involved with *in vivo* systems as well as the *in vitro* assays. One caveat is that we tested the adjuvants in BALB/c mice which are more prone to Th2 responses (41) and **2G272** may not skew toward a Th2 effect in all genetic backgrounds (e.g., C57BL/6) (42). In a separate series of experiments using a different antigen there was a predominance of antigen specific IgG1 production in C57BL/6 mice using **2G272** as a single adjuvant (31). However, **2G272** had an acceptable safety profile with minimal systemic elevation of inflammatory markers unlike ionomycin.

## 5 Conclusion

We present a small-molecule adjuvant, **2G272** that enhances APC function by triggering SOCE dependent intracellular Ca^2+^ influx, upregulating costimulators CD80 and CD86 on the surface and on the EVs from released from activated APCs, and results in augmented T-cell proliferation. In a murine preclinical model, **2G272** adjuvanted ovalbumin promoted Th2-dominant immune responses with a promising early safety profile.

## Data Availability

The original contributions presented in the study are included in the article/**Supplementary Material**. Further inquiries can be directed to the corresponding authors.
