# Supplementary material for: Application of a small molecule calcium influx inducer as a vaccine adjuvant: enhancing Th2-biased immune responses
Source: Front Immunol. 2026 Feb 17;17:1704416. doi: 10.3389/fimmu.2026.1704416 (PMC12953538; doi:10.3389/fimmu.2026.1704416)
Supplement: Supplementary file 1 [file DataSheet1.docx]

Supplementary Material

**
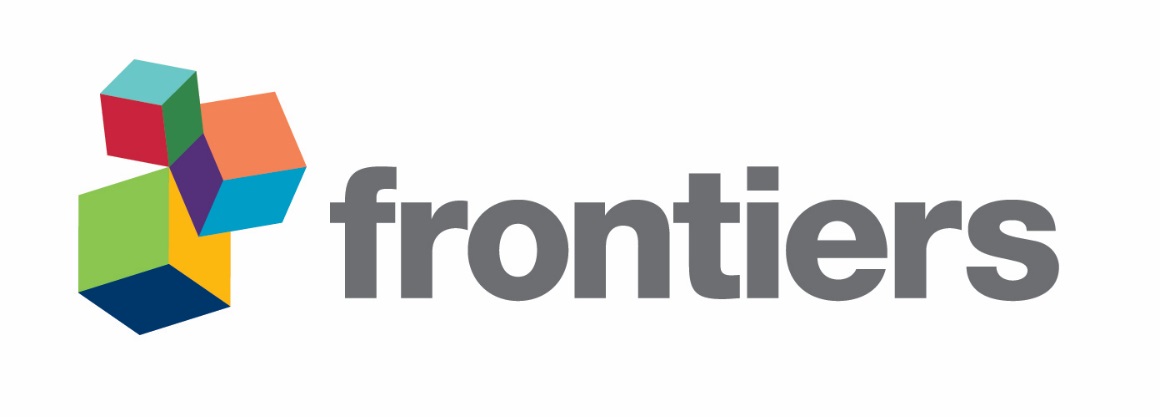
**

**
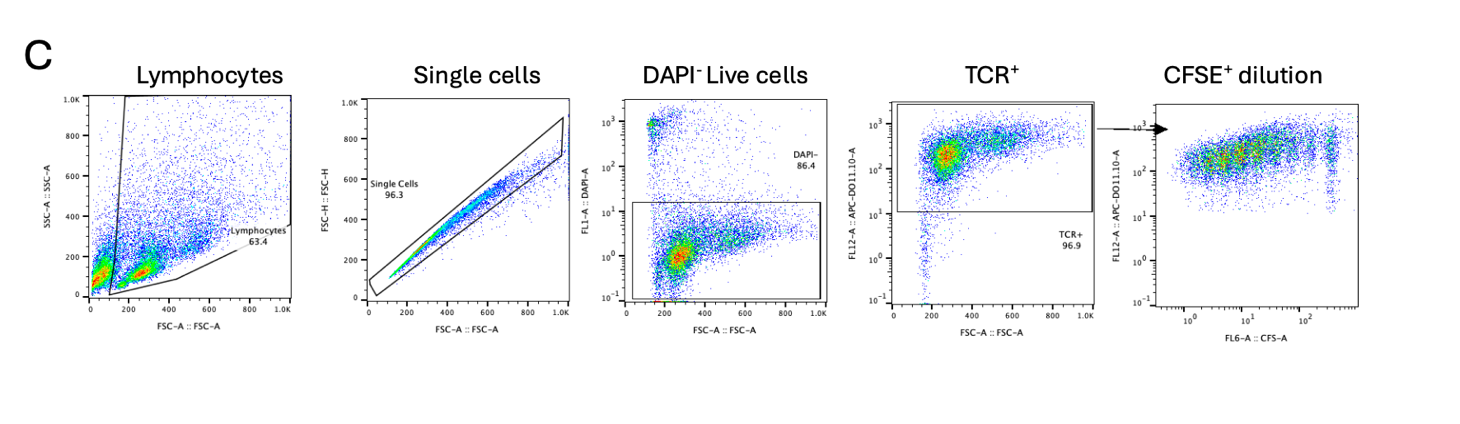
Supplementary Figures**

**Supplementary Figure S1.** **Gating strategies used for the flow cytometric analyses.**

1. The gating strategy used for MFIs of costimulatory molecules on CD11c positive cells. Flow cytometry data were gated to distinguish lymphocytes and singlets based on forward and side scatter. DAPI ^high^ dead cells were excluded from the analysis. Then CD11c^+^ cells were gated, and MFIs of costimulatory molecules on CD11c^+^ cells were calculated.
2. The gating strategy used for T cell proliferation assay. In DAPI^low^ live cells, CD4-positive cells were gated. Cell proliferation was monitored by CFSE dilution. Percentages of divided cells relative to the original population were calculated.
3. The gating strategy used for T cell proliferation assay with EVs. In DAPI^low^ live cells, DO11.10-positive cells were gated. Cell proliferation was monitored by CFSE dilution Percentages of divided cells relative to the original population were calculated.

*
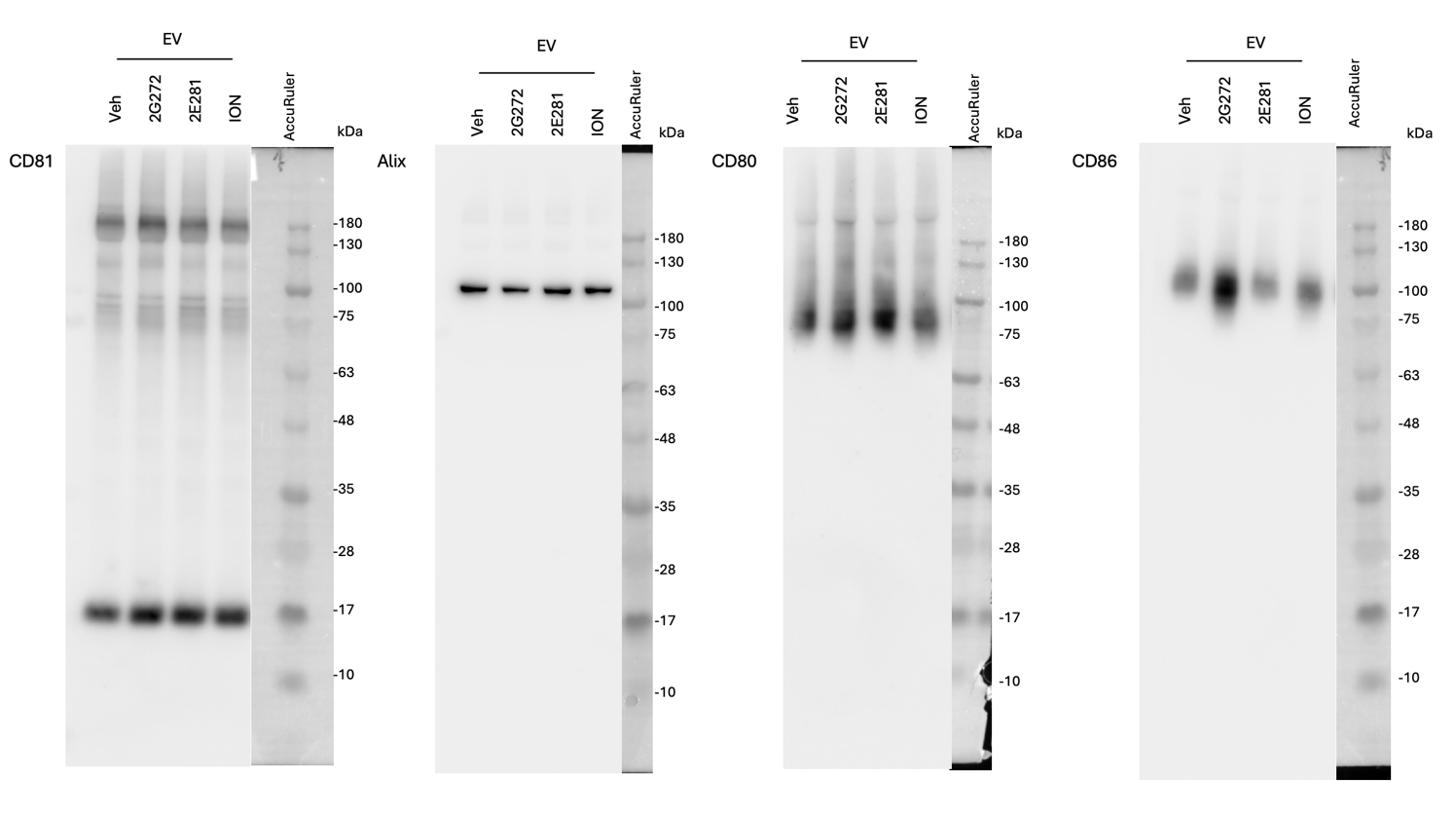
*

**Supplementary Figure S2. Original immunoblots from Figure 4A**.

**Supplementary Figure S3. Morphological analysis of EV_2G272_ and EV_Veh_**.

Shown are the morphologies of EVs released from **2G272**- or vehicle-treated mBMDC which were visualized using CryoTEM by the Nanoparticle Characterization Laboratory.

**Supplementary Table S1. Reagents used in experiments**

| **Reagents** | **Source** | **Catalog #** |
| --- | --- | --- |
| MPLA | InvivoGen | tlrl-mpls |
| PMA | Invivogen | tlrl-pma |
| Ionomycin | Tocris | 1704 |
| BTP2 (YM-58483) | Tocris | 3939 |
| MF59 | InvivoGen | vac-adx-10 |
| OVA | Worthington Biochemical | LS003056 |
| OVA_323-339_ (ISQAVHAAHAEINEAGR) | AnaSpec | AS-27024 |
| 1 x PBS | Thermo Fisher Scientific | 14190 |
| GM-CSF | BioLegend | 576308 |
| **Cell culture** | | |
| RPMI 1640 | Thermo Fisher Scientific | 11875 |
| Heat-inactivated FBS | Omega Scientific | FB-12 |
| Penicillin, Streptomycin | Thermo Fisher Scientific | 25-512 |
| 2-mercaptoethanol | Millipore Sigma | M7522 |
| **EV isolation** |  |  |
| Exosome depleted FBS | Thermo Fisher Scientific | A27208 |
| 31.5 mL open-top polypropylene UC tube | Beckman Coulter Life Sciences | 358126 |
| **T cell proliferation assay** | | |
| EasySep Mouse CD4^+^ T cell isolation kit | STEMCELL Technologies | 19852 |
| CFSE | Thermo Fisher Scientific | C34554 |
| **Calcium Influx assay** | | |
| Fura-8-AM | ATT Bioquest | 21055 |
| 1 x HBSS | Thermo Fisher Scientific | 14175 |
| Pluronic F127 | Thermo Fisher Scientific | P3000MP |
| **Immunoblotting** | | |
| PhosphoSafe extraction reagent | MilliporeSigma | 71296 |
| Protease inhibitor cocktail | Roche | 11697498001 |
| Micro BCA Assay Kit | Thermo Fisher Scientific | 23235 |
| BCA Assay kit | Thermo Fisher Scientific | 23225 |
| NuPAGE sample buffer | Thermo Fisher Scientific | NP007 |
| NuPAGE 4-12% Bis-Tris Gels | Thermo Fisher Scientific | NP0329 |
| Immobilon-P PVDF membranes | MilliporeSigma | IPVH00010 |
| ProSignal Dura ECL | Thermo Fisher Scientific | 34076 |
| AccuRuler Prestained Protein Ladder | Lamda Biotech | G02101 |
| **Cytokine ELISA** | | |
| Mouse IFN-γ Duo Set | R&D systems | DY485 |
| Mouse IL-2 Duo Set | R&D systems | DY402 |
| alpha-1 acid glycoprotein Simple Step ELISA kit | Abcam | Ab264605 |
| Mouse IL-5 antibody | BD Pharmingen | 554393 |
| Biotinylated rat anti-mouse IL-5 antibody | BD Pharmingen | 55397 |
| mouse IL-5 standard | BD Pharmingen | 554581 |
| Half area 96 well ELISA plate | Coring | 3690 |
| ProcartaPlex^TM^ Multiplex Immunoassay | Thermo Fisher | PPX-06-MXKA4FF |
| **Immunoglobulin ELISA** | | |
| Influenzas A H1N1 (A/California/04/2009) Hemagglutinin / HA Protein (His Tag) | Sino Biological | 11055-V08B |
| IgG1-AP goat anti-mouse | Southern Biotech | 1070-04 |
| IgG2a-AP goat anti-mouse | Southern Biotech | 1080-04 |
| p-Nitrophenyl Phosphate tablets (pNPP) | Sigma | N2770 |

**Supplementary Table S2. Antibodies used in these experiments**

| **Antibody (clone)** | **Dilution Factor** | **Source** | **Catalog #** |
| --- | --- | --- | --- |
| **Immunoblotting** | | | |
| Anti-CD81 (D5O2Q) | 1000 | Cell Signaling | 10037 |
| Anti-Alix (3A9) | 1000 | Cell Signaling | 2171 |
| Anti-CD86 (E5W6H) | 1000 | Cell Signaling | 19589S |
| Anti-CD80 (E6J6N) | 1000 | Cell Signaling | 54521 |
| Anti-Rabbit IgG, HRP | 5000 | Cell Signaling | 7074 |
| Anti-mouse IgG, HRP | 5000 | Cell Signaling | 7076 |
| **Flow cytometry** | | | |
| Anti-mouse DO-11.10 clonotypic TCR, AF647 (KJ1-26) | 300 | BD Biosciences | 562524 |
| Anti-mouse CD4 | 600 | ThermoFisher | 17-0042-82 |
| Staining Buffer |  | BD Biosciences | 554657 |

## Supplementary Table S3. Limit of detection (LOD) for ELISAs used in this study

| Reagents | Limit of detection |
| --- | --- |
| Mouse IL-5 | 30 ~ 40,000 pg/mL |
| Mouse IL-12 | 4 ~ 30,000 pg/mL |
| Mouse IFNγ | 15.6 ~ 4,000 pg/mL |
| Mouse IL-2 | 8 ~ 2,000 pg/mL |
| Mouse CXCL10 | 2 ~ 8,000 pg/mL |
| Mouse IL6 | 47 ~ 48,200 pg/mL |
| Mouse IL12 | 3 ~ 13,400 pg/mL |
| Mouse CXCL1 | 30 ~ 7,800 pg/mL |
| Mouse CCL5 | 20 ~ 20,200 pg/mL |
| Mouse TNFa | 20 ~ 20,600 pg/mL |
| Mouse CCL2 | 13 ~55,000 pg/mL |
| Alpha-1-acid glycoprotein | 2 ~ 100 µg/mL |
| OVA IgG1-ELISA | 68 〜61×10^7^U/mL |
| OVA IgG2a-ELISA | 2.3 〜 21 × 10^6^ U/mL |
| OVA IgG-ELISA | 75 〜 67× 10^7^ U/mL |
